# Rank Order of Small Molecule Induced Hypoxiamimesis to Prevent Retinopathy of Prematurity

**DOI:** 10.3389/fcell.2020.00488

**Published:** 2020-06-23

**Authors:** George Hoppe, Youstina Bolok, Leah McCollum, Jin Zhang, Jonathan E. Sears

**Affiliations:** ^1^Ophthalmic Research, Cole Eye Institute, Cleveland Clinic, Cleveland, OH, United States; ^2^Cardiovascular and Metabolic Sciences, Lerner Research Institute, Cleveland Clinic, Cleveland, OH, United States

**Keywords:** hypoxia inducible factor (HIF), retinopathy of prematurity (ROP), hyperoxia and hypoxia, prolyl hydroxylase (PHD), prolyl hydroxylase (PHD) inhibitor, oxygen induced retinopathy

## Abstract

Here we rank order small molecule inhibitors of hypoxia inducible factor (HIF) prolyl hydroxylases (PHDs) using severity of oxygen induced retinopathy (OIR) as an outcome measure. Dose response analyses in cell cultures of hepatoma (Hep3B), retinal Müller cells (MIO-M1) and primary retinal endothelial cells were conducted to evaluate potency by comparing dose to HIF-1,2 protein levels by western blotting. *In vivo* dose response was determined using the luciferase-transgene HIF reporter (luc-ODD). Each compound was placed in rank order by their ability to reduce neovascularization and capillary drop out in the OIR mouse model. An *Epas1* KO confined to retinal Müller cells was used to determine whether successful protection by HIF stabilization requires HIF-2. Two candidate small molecules can prevent OIR by stabilizing HIF-1 to prevent oxygen induced growth attenuation and vascular obliteration. Müller cell HIF-2, the mediator of pathologic retinal angiogenesis, is not required for protection. The lack of dependence on Müller cell HIF-2 predicts that inhibition of HIF PHD will not drive pathological angiogenesis.

## Introduction

Survival after premature birth requires oxygen supplementation that is simultaneously harmful to premature developing tissues (Park et al., 2010). The normal oxygen saturation of a fetus *in utero*, measured at 85–93%, correlates with mean partial pressure of oxygen (PaO_2_) of 53 mmHg, and contrasts sharply with accepted target saturations of 91–95% that create a mean arterial PaO_2_ close to 107 mmHg, which is well beyond the toxic saturation of 83 mm Hg (Castillo et al., 2008; Owen and Hartnett, 2014). The oxygen-induced growth inhibition of vascular tissues creates retinopathy of prematurity (ROP), a vasoproliferative disease that blinds 170,000 infants annually worldwide (Hartnett and Penn, 2012). Recent randomized clinical trials have tested static low oxygen targets to static high oxygen targets and determined that lower oxygen targets do indeed reduce ROP but unfortunately increase mortality (SUPPORT Study Group of the Eunice Kennedy Shriver NICHD Neonatal Research Network et al., 2010).

The tension between survival and acquired, oxygen-induced defects in multiple organ systems such as the brain, eye, lung, and kidney stimulated the idea that the origin of oxygen induced pathology was the down regulation of an oxygen sensitive transcription factor, hypoxia inducible factor (HIF), that regulates the expression of cytoprotective and angiogenic genes (Semenza et al., 1991; Semenza and Wang, 1992; Semenza, 1998). Experiments that revealed the central role of vascular endothelial growth factor in the development of oxygen-induced retinopathy (OIR) simultaneously provided a platform for prevention of ROP (Aiello et al., 1994; Aiello et al., 1995); VEGF supplementation during the hyperoxic phase 1 of ROP prevented vascular obliteration and retinovascular growth attenuation, and thereby removed the substrate for ROP by permitting growth even in hyperoxia (Alon et al., 1995; Pierce et al., 1996). VEGF expression was soon found to be regulated by HIF (Forsythe et al., 1996). These discoveries led to the hypothesis, proven in two species, that activating HIF (hypoxiamimesis) during hyperoxia could prevent ROP (Sears et al., 2008; Trichonas et al., 2013).

HIF is an α/β heterodimeric transcription factor that regulates expression of many genes in addition to VEGF that are related to adaptive responses to hypoxia, such as erythropoietin and multiple metabolic enzymes relevant to enhancing glycolysis. The stability and hence transcriptional activity of HIF is regulated by hydroxylation of Pro-402 or Pro-564 within the C-terminal oxygen dependent degradation of HIFα (For review, see, Dann and Bruick, 2005; Hirota and Semenza, 2005; Kaelin, 2005; Schofield and Ratcliffe, 2005). Hydroxylation is catalyzed by 2-oxoglutarate (2-OG) dependent dioxygenases (PHD 1,2, or 3) as well as an asparaginyl hydroxylase (factor inhibiting HIF, FIH), enabling binding of an E3 ubiquitin ligase, the von Hippel Lindau protein (pVHL), that ubitquinates HIFα to target it to the proteosome (Epstein et al., 2001; Ivan et al., 2001; Hewitson et al., 2002). The requirement of oxygen as a cofactor to trans-4-polyl hydroxylation makes HIF PHDs a cellular oxygen sensor. One oxygen from dioxygen is used to hydroxylate proline. The remaining oxygen is incorporated into succinate during oxidative decarboxylation of 2-OG. Cofactors to this reaction are Fe2+, 2-OG, ascorbate, and molecular dioxygen. Activation or stabilization of HIF can be achieved pharmacologically by inhibiting HIF PHD using analogs of 2-OG (Mole et al., 2003). Small molecule analogs such as carbonyl glycines can competitively inhibit the PHD – even during hyperoxia – to stabilize HIFα so that it can translocate to the nucleus, bind HIFβ to make the active mature heterodimer. We have definitively demonstrated that systemic HIF stabilization can induce protection against OIR and bronchopulmonary dysplasia in two different species (Sears et al., 2008; Trichonas et al., 2013).

This protection was induced by two different small molecules, each with a carbonyl glycine structure. The first, dimethyloxalylglycine (DMOG), is a non-selective prolylhydroxylase inhibitor that is very unstable. *In vivo*, DMOG is broken down quickly in the first pass through the liver into n-oxalylglycine and methyl oxalylglycine (Olenchock et al., 2016). Because the latter two species are cell membrane impermeable, they do not enter the circulation in sustainable concentrations and hence only have biologic activity in the liver. In contrast, Roxadustat, a second carbonyl glycine with an isoquinolone structure containing a phenyl ester is more stable *in vivo*. Therefore, Roxadustat can target the liver but also retina, kidney, and brain (Hoppe et al., 2016). Although a hepatic specific HIF-1α knockout prevents protection by DMOG, to a large degree Roxadustat circumvents the liver by directly targeting the retina (Hoppe et al., 2014, 2016).

Smith and Penn originally created experimental correlates of ROP in the mouse and the rat, respectively, termed OIR and the rat 50/10% model (Penn and Thum, 1989; Smith et al., 1994). We have evaluated the strategy of stabilizing HIF during hyperoxia using protection against vascular obliteration and pathological angiogenesis measured in retinal flatmounts as one metric of efficacy. In retinas protected against retinopathy we find (1) no liver or kidney toxicity, (2) 85–100% reduction of oxygen induced retinal vasoobliteration and neovascularization in mice and rats, (3) normalization of the electroretinogram in hyperoxia, (4) normalization of ocular coherence tomography, (5) a cadre of serum biomarkers secreted by liver, and (6) and a metabolic switch to upregulate the urea cycle and 1C/serine metabolism (Hoppe et al., 2014, 2016; Singh et al., 2019, 2020). We observed that when using DMOG, hepatic HIF-1 was necessary and sufficient to prevent OIR but that Roxadustat could partially overcome hepatic HIF-1 ablation because it also targeted the retina. Results of these experiments offer the possibility that low dose, intermittent application of small molecule antagonists to HIF PHD such as Roxadustat could be administered to susceptible infants because the liver acts synergistically with the retina. A brief stabilization of HIF could direct a transcriptional stimulus that might persist for days to permit the liver to protect multiple organ systems from oxygen induced growth attenuation and vascular obliteration.

In order to determine which small molecule stabilizer would be best to translate to the clinic, here we rank order seven small molecule HIF stabilizers to determine which is best at mediating protection against OIR in the mouse. Three groups of drugs were tested: DMOG, Roxadustat, AK9 (known carbonyl glycines), as well as AR0, AR2, AR4, and AX1. In addition, we demonstrate that the mediator of pathologic angiogenesis, HIF-2, is not required for protection.

## Results

### AK9 Is a Potent Epo Inducer

**FIGURE 1:**
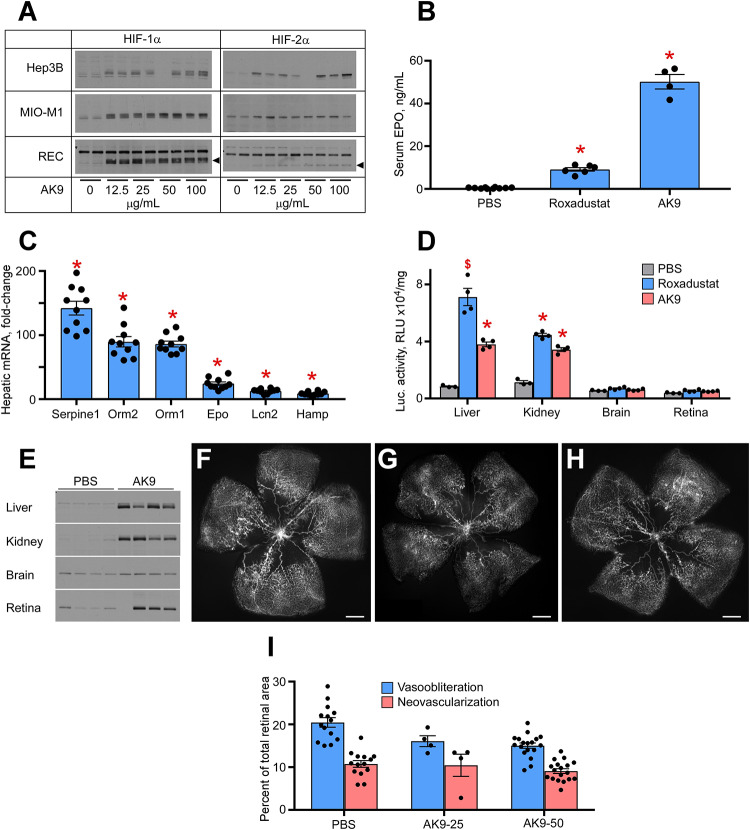
Potency of AK9 in HIF stabilization and prevention of OIR. **(A)** Western blot analysis of HIF-1α/2α protein levels in three types of cultured cells treated overnight with the indicated concentrations of AK9. **(B)** Levels of serum Epo obtained from blood of P8 pups 6 h after a single intraperitoneal injection of PBS (control), 10 μg/g Roxadustat or 50 μg/g AK9. PBS, *n* = 10; Roxadustat, *n* = 6; AK9, *n* = 4. **(C)** Hepatic mRNA levels of select genes, known to be upregulated by Roxadustat (Hoppe et al., 2016). P8 mouse pups were administered 50 μg/g AK9 by intraperitoneal injection, euthanized 6 h later and their livers were dissected for mRNA analysis as described in Methods. Gene expression levels are presented as fold-change vs control (PBS injection = 1), *n* = 10 for each experimental condition. **(D)** Luciferase activity in tissue homogenates of P8 Luc-ODD mouse pups 3 h after PBS (control), Roxadustat (10 (μg/g) or AK9 (50 μg/g) injections serving as a reporter of HIF stability. Data expressed as relative light units (RLU) normalized to tissue protein. BS, *n* = 3; Roxadustat *n* = 4; AK9, *n* = 4. **(E)** Western blot analysis of HIF-1(α protein levels in the organs of P8 mouse pups 3 h after PBS (control) or AK9 (50 μg/g) injections. **(F–H)** Representative retinal flatmounts of the control PBS-injected **(G)** and AK9-treated **(H)** mouse pups at P17 following hyperoxia exposure from P7 to P12 as detailed in Methods, scale bar = 500 μm. **(I)** Quantitative analysis of retinal flatmount images exemplified in panels **(G,H)** for areas of capillary loss (vasoobliteration) and vascular tufting (neovascularization) tested at two doses of AK9, 25 and 50 μg/g. PBS, *n* = 14; AK9-25, *n* = 4; AK9-50, *n* = 18. All *p* values vs PBS were >0.05. *P* values are presented as follows, * ≤ 0.0001; $ ≤ 0.005; & ≤ 0.01; # ≤ 0.05, and represent statistical difference vs PBS control.

Figure 1A demonstrates a dose response analysis of AK9 in cultured hepatocytes (Hep3B), retinal Müller cells (MIO-M1), and retinal endothelial cells (RECs). When compared to DMOG and Roxadustat, AK9 stabilized HIF-1α and HIF-2α in cultured hepatocytes to a greater degree than in Müller cells or RECs. At 6h post intraperitoneal injection, AK9 pups demonstrated a 5-fold increased serum Epo concentration when compared to Roxadustat (Figure 1B). AK9 also had a similar profile to Roxadustat in terms of hepatokine expression. Previous experiments had determined the top hits after Roxadustat and DMOG injection in mice (Hoppe et al., 2016); AK9 demonstrated a nearly identical expression pattern (Figure 1C). Using the luciferase oxygen dependent degradation domain transgenic mouse (luc-ODD) (Safran et al., 2006), we were able to examine organ specific HIF stabilization, which definitively demonstrated the strong trophism of AK9 for the liver in comparison to Roxadustat (Figure 1D). Western blot analysis directly confirmed organ-specific stabilization of HIF-1α (Figure 1E). AK9 was only modestly effective at preventing OIR, however, using 25 and 50 μg/g weight of a pup (Figures 1F–H). Increasing dose of AK9 does not enhance protection (Figure 1I). The experiments in Figure 1 demonstrate that AK9 stabilizes HIF-2α better than Roxadustat in hepatocytes, but does not stabilize HIF2α in Muller cells. Organ specific western blots and gene reporter assessing PHD inactivation demonstrate that liver, and to a lesser degree kidney, are the main targets of AK9.

### AR0 and Roxadustat Are Equivalent in Preventing OIR

**FIGURE 2:**
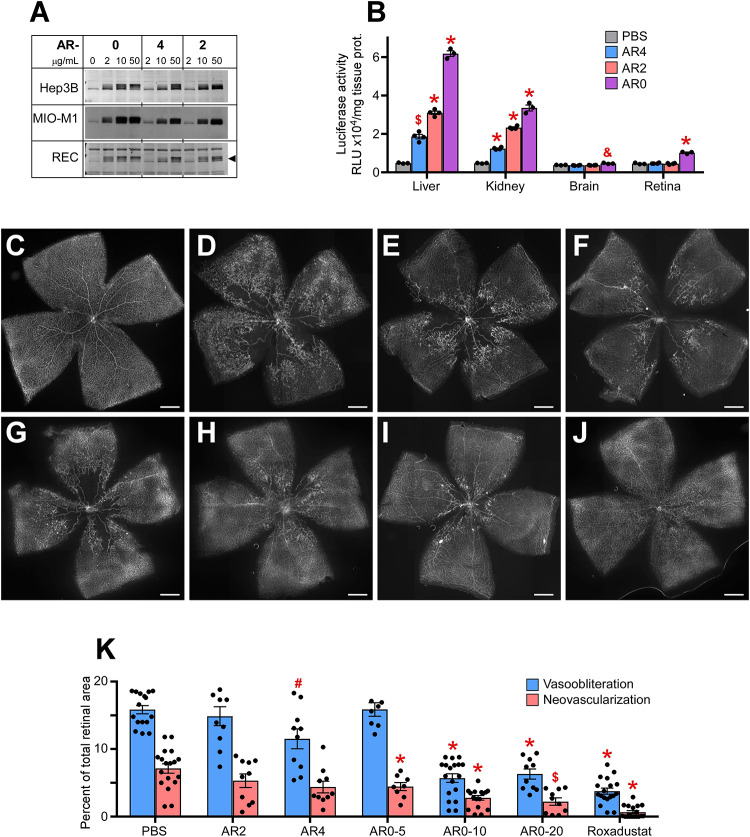
Potency of AR compounds in HIF stabilization and prevention of OIR. **(A)** Western blot analysis of HIF-1α protein levels in three types of cultured cells treated overnight with the indicated concentrations of AR0, AR4 or AR2. **(B)** Luciferase activity in tissue homogenates of P8 Luc-ODD mouse pups 3 h after injections of PBS (control), AR4 (40 μg/g), AR2 (40 μg/g) or AR0 (20 μg/g) expressed as RLU/mg tissue protein. PBS, *n* = 3; AR4, *n* = 5; AR2 = 5: AR0, *n* = 4. **(C–J)** Representative retinal flatmounts of the naïve room air reared **(C)** or hyperoxia-challenged **(D–J)** P17 mouse pups according to the OIR protocol, who were treated with PBS **(D)**, 40 μg/g AR4 **(E)**, 40 μg/g AR2 **(F)**, 5 μg/g AR0 **(G)**, 10 μg/g AR0 **(H)**, 20 μg/g AR0 **(I)** or 10 μg/g Roxadustat **(J)**. Scale bar = 500 μm. **(K)** Quantitative analysis of retinal flatmount images shown in panels **(D–J)** for areas of capillary loss (vasoobliteration) and vascular tufting (neovascularization). PBS, *n* = 16; AR2, *n* = 10; AR4, *n* = 10; AR0-5, *n* = 8; AR0-10, *n* = 18; AR0-20, *n* = 10; Roxadustat, *n* = 18. *P* values are presented as follows, * ≤ 0.0001; $ ≤ 0.005; & ≤ 0.01; # ≤ 0.05, and represent statistical difference vs PBS control.

We next examined a second class of drugs designated AR0, AR4, and AR2. All three were potent stabilizers of HIF-1α in Muller cells, RECs, and hepatocytes (Figure 2A). When comparing organ specific stabilization using the luc-ODD transgenic mouse, AR0 induced the highest luminescence in liver and retina (Figure 2B). When each compound was tested in the OIR model, of the ARs, AR0 performed best when comparing amount of vasoobiteration and neovascularization (representative flatmount images Figures 2C-G) at 20 μg/g. This dose was further refined to show near equal efficacy to Roxadustat and better efficacy than DMOG using 10 μg/g weight of pup, injected at P6, P8, and P10 as is the standard for these compounds (Figures 2H–J). All doses are quantified in Figure 2K. The experiments in Figure 2 demonstrate that the ARs are potent HIF-1,2α stabilizers that, like other small molecule HIF PHD inhibitors that are administered systemically, target primarily liver and kidney. Although AR0 was most effective at preventing capillary drop out, all the ARs were effective at reducing neovascularization.

**FIGURE 3:**
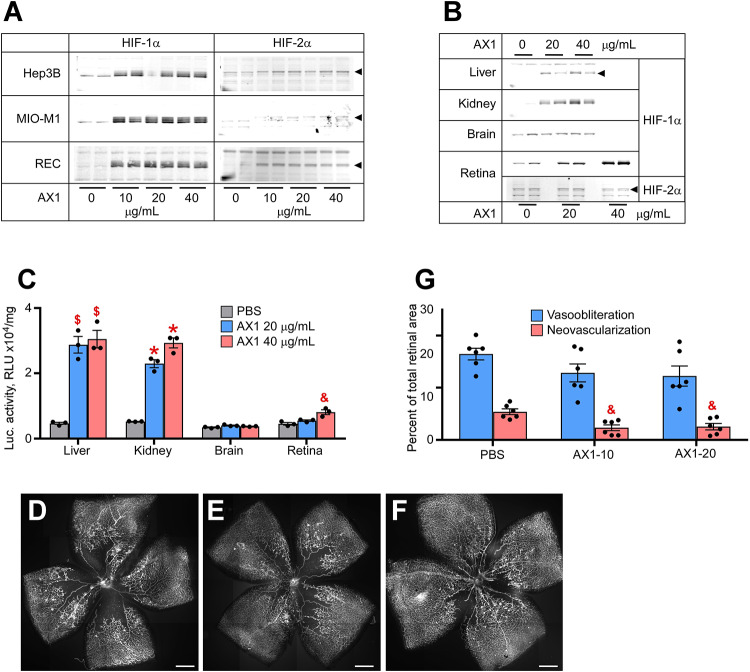
Potency of AX1 in HIF stabilization and prevention of OIR. **(A)** Western blot analysis of HIF-1α/2α protein levels in three types of cultured cells treated overnight with the indicated concentrations of AX1. **(B)** Western blot analysis of HIF-1α/2α protein levels in the organs of P8 mouse pups 3 h after injections with PBS (control) or indicated doses of AX1. **(C)** Luciferase activity in tissue homogenates of P8 Luc-ODD mouse pups 3 h after injections of PBS (control) or indicated doses of AX1, *n* = 3 for each experimental condition. **(D–F)** Representative retinal flatmounts of the P17 mouse pups subjected to the OIR model and treated with PBS **(D)**, 10 μg/g (E) or 20 μg/g **(F)** of AX1, scale bar = 500 μm. **(G)** Quantitative analysis of retinal flatmount images represented in panels **(D–F)** for areas of vasoobliteration and neovascularization, *n* = 6 for each experimental condition. *P* values are presented as follows, * ≤ 0.0001; $ ≤ 0.005; & ≤ 0.01; # ≤ 0.05, and represent statistical difference vs PBS control.

The last compound tested was AX1. Figure 3A demonstrates potent HIF stabilization in hepatocytes, Muller cells, and RECs. AX1, like AK9 was distinctly novel from all other compounds in that it did not stabilize HIF-2α in cultured Muller cells. Figure 3B shows organ specific endogenous HIF stabilization notable for the preference of kidney over liver and brain. Figure 3C quantifies HIF stabilization using the luc-ODD transgenic reporter mouse; as before with Muller cells, AX1 did not induce HIF-2α in total retina. In terms of reducing avascular retina, AX1 had a small effect (Figures 3D–F). However, the effect on neovascularization was equal to Roxadustat and DMOG (Figure 3G). This was particularly interesting because it reduced pathological angiogenesis even when ischemia persisted.

### Rank Order of all Compounds Using OIR as an Outcome Measure

**FIGURE 4:**
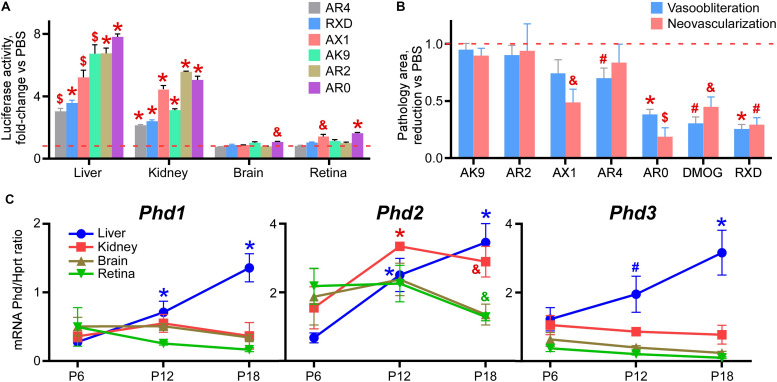
Rank order of the tested drug-like compounds. **(A)** Luciferase activity in tissue homogenates of P8 Luc-ODD mouse pups 3 h after injections of AR4 (40 μg/g), Roxadustat (RXD, 10 μg/g), AX1 (40 μg/g), AK9 (40 μg/g), AR2 (40 μg/g) or AR0 (20 μg/g) plotted as fold-change vs control PBS injection (red dashed line). Number of replicates is given in Figures 1–3. **(B)** Cumulative quantitative analysis of all retinal flatmount images exemplified in Figures 1–3 presented as a drug-induced reduction in avascular or neovascular retinal area from the control PBS treatment (red dashed line). Number of replicates is given in Figures 1–3. **(C)** Organ-specific developmental expression of *Phd1*, *Phd2*, and *Phd*3 plotted as corresponding mRNA levels normalized to mRNA levels of *Hprt*. Color of the *p* value marker corresponds to color of the line. *n* = 6 for each experimental condition and each gene.

Figure 4A demonstrates the overall rank grouping of these agents in terms of organ specific luciferase activity in the Luc-ODD transgenic mouse. Figure 4B rank orders all of the compounds in regard to reducing vaso-obliteration and neovascularization, normalized to PBS. We observed that AR0 was as effective as DMOG and Roxadustat in reducing vasoobliteration and neovascularization.

There are three isoforms of prolyl hydroxylases (PHD-1,2,3) that are active in down regulating HIF. Figure 4C demonstrates the developmental expression of all PHDs in P8-P17 mouse pups in liver and retina. The earliest expressor is PHD-1, which gives way to PHD-2, especially in liver.

### HIF-2 Is Not Required for Roxadustat Induced Vasoprotection

The perplexing finding that AX1 was very effective at preventing neovascularization but not effective at preventing vasoobliteration, in conjunction with lack of HIF-2α induction in Müller cells, suggested that HIF stabilization might need intact HIF-2α in Müller cells. Müller cells are reported to regulate neovascularization and therefore it is of great value to determine whether HIF stabilization therapeutic strategy requires an active HIF-2α, to determine whether effective hypoxiamimesis (prevention of OIR) requires the action of a gene associated with pathologic neovascularization. Thus, to test whether hypoxiamimesis requires HIF-2α for preventing OIR, and hence might worsen neovascular retinopathy, we conditionally ablated HIF-2α from Müller cells, and compared the retinal phenotype in the OIR model with and without Roxadustat.

**FIGURE 5:**
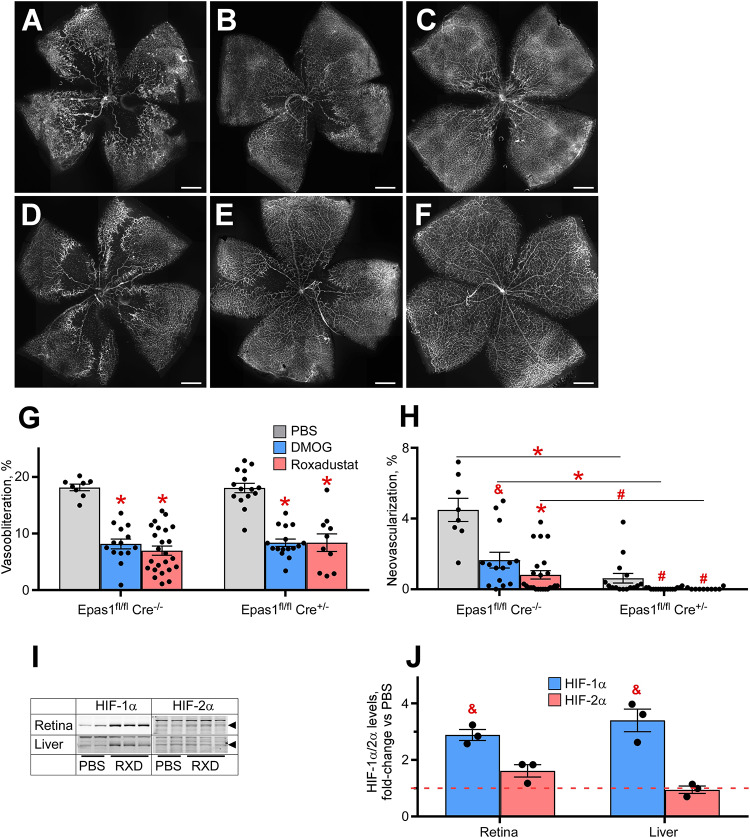
Effect of HIF-2α deletion from the retinal Müller cells on drug-induced HIF stabilization and OIR prevention. **(A–F)** Representative retinal flatmounts of the P17 wild-type **(A–C)** or Müller cell-specific HIF-2α KO **(D–F)** mouse pups subjected to hyperoxia from P7 to P21 and thrice injected with 200 μg/g DMOG **(B,E)** or 10 μg/g Roxadustat **(C,F)** at P6, P8 and P10. Scale bar = 500 μm. **(G,H)** Quantification of the retinal capillary loss area [vasoobliteration, **(G)**] and vascular tufts area [neovascularization, **(H)**] in retinal flatmount images demonstrated in panels **(A–F)**. Epas1^fl/fl^:Cre^–/–^, PBS, *n* = 8; DMOG, *n* = 14; Roxadustat, *n* = 24. Epas1^fl/fl^:Cre^±^, PBS, *n* = 15; DMOG, *n* = 16; Roxadustat, *n* = 10. **(I)** Western blot analysis of HIF-1α/2α protein levels in the liver and retina of the P8 Müller cell-specific HIF-2α KO mouse pups 3 h after injections with PBS or 10 μg/g Roxadustat (RXD). **(J)** Semi-quantitative analysis of HIF-1α and HIF-2α protein levels by densitometry of the western blotting images shown in panel **(I)** plotted as fold-change vs PBS injection (red dashed line), *n* = 3 for each experimental condition. *P* values are presented as follows, * ≤ 0.0001; $ ≤ 0.005; & ≤ 0.01; # ≤ 0.05, and represent statistical difference vs PBS control, unless otherwise indicated as in panel **(H)**.

Figures 5A–F demonstrates retinal flat mounts of wild type and Müller cell HIF-2α knockout (KO) mice treated with PBS, DMOG or Roxadustat. The top panels (Figure 5A control sham injection; Figures 5B,C DMOG vs Roxadustat, respectively) compare Roxadustat to DMOG in the wild type mouse. DMOG and to a larger extent Roxadustat prevent OIR, and this effect is maintained by these carbonyl glycines in Müller cell HIF-2α KO mice (Figure 5D, sham injection, Figure 5E, DMOG, and Figure 5F Roxadustat). Quantification of oxygen induced vaso-obliteration (Figure 5G) and pathologic angiogenesis (Figure 5H) demonstrates protection in both the WT and KO mice. A western blot in Figure 5I, quantified by densitometry in Figure 5J, further demonstrates that systemic Roxadustat primarily upregulates HIF-1α when compared to HIF-2α using liver and retina lysates (Fig. 5J).

## Discussion

We have definitively demonstrated that small molecules can be administered systemically to prevent retinal and lung disease associated with hyperoxia of preterm birth. These drugs target both the liver and eye synergistically. This allows a low, intermittent dose capable of protecting distal capillary beds while reducing the chance for toxicity (Hoppe et al., 2016).

Our studies demonstrate that there are at least two small molecule drug candidates that reduce pathologic angiogenesis by reducing avascular retina. The first, Roxadustat, is in phase 3 clinical trials to treat anemia of chronic kidney disease (Martin et al., 2017; Chen et al., 2019; Sanghani and Haase, 2019). The second, AR0, is in preclinical development to prevent ROP. Roxadustat is a carbonyl glycine, whereas AR0 is a benzolamide constructed through modeling of the PHD2 oxoglutarate binding site (Rabinowitz, 2013). Both small molecules targeted PHD2 more effectively than PHD1 or 3. The developmental time course of retinal PHD2 expression in retina becomes dominant at P8 at just the time when HIF PHD inhibition should be maximal to get the best protection. The weaker induction of HIF-2 was seen with both lead compounds; our HIF-2α KO experiments demonstrate that HIF-2α expression is not necessary for protection by HIF stabilization, whereas HIF-2α induction in hepatocytes is necessary for Epo upregulation (Kapitsinou et al., 2010; Minamishima and Kaelin, 2010; Haase, 2013; Koury and Haase, 2015; Imeri et al., 2019), a critical feature to increasing red blood cell density. Of the small molecules tested here, AK9 is the most robust Epo inducer, at least *in vivo*.

The cadence of ROP is predictable. Generally, the earliest stages can be seen at 30 weeks corrected gestational age (CGA) no matter what the CGA at birth. This suggests that a clinical trial might administer the molecules intravenously, beginning at birth, continuing at 4–7 day intervals until 30 weeks, thereby inducing normal, sequential retinovascular growth to deprive ROP of its substrate, the avascular retina, in phase 2.

In the retina, pathologic angiogenesis is associated with Müller cell HIF-2α expression (Weidemann et al., 2010; Becker et al., 2018). Here we show that ablation of HIF-2α does not lessen the effect of HIF stabilization in the prevention of experimental ROP, indicating that HIF-2α stabilization is not required for DMOG or Roxadustat induced protection. Thus, Roxadustat might be safe to use in diabetics with anemia of CKD and simultaneous diabetic retinopathy because Roxadustat does not require Müller cell HIF-2, the mediator of pathological angiogenesis, to induce protection of retinal vessels, and Roxadustat has a weak effect on Müller cell HIF-2α. These findings provide a molecular mechanism to support the use of HIF PHD inhibitors in the treatment of diabetic patients with anemia of chronic kidney disease or during hyperoxia in severely premature infants at risk for ROP, a leading cause of childhood blindness.

In conclusion, we find two candidate small molecules that are effective in preventing OIR, which is the experimental correlate of ROP. Second, stabilization of Müller cell HIF-2α is not necessary for protection. Finally, AK9 is a potent inducer of erythropoietin.

## Materials and Methods

All animal experiments were approved by Institutional Animal Care and Use Committee, Cleveland Clinic, and in accordance with the ARVO statement for the use of animals in ophthalmic and vision research.

### HIF Stabilizers Sources and Formulation

Dimethyloxalylglycine was purchased from Frontier Scientific (Logan, UT, United States) and Roxadustat (FG-4592) from Adooq Bioscience (Irvine, CA). AK9, AR0, AR4, AR2 and AX1 were generously provided by Akebia Therapeutics (Cambridge, MA, United States). Dimethyloxalylglycine was dissolved directly in PBS at concentration of 20 mg/mL. Roxadustat was fist dissolved in DMSO at 50 mg/mL then further dissolved in PBS to bring it to 1 mg/mL. Akebia compounds were first dissolved in 20% 2-hydroxyprolyl-β-cyclodextrin (Cayman Chemical, Ann Arbor, MI, United States) at 50 mg/mL then further diluted in PBS to achieve desirable doses for injections. Volumes of injections were always kept at 10 μL per gram of body weight. Likewise, all volumes of drugs added to cell cultures were formulated at 10 μL per milliliter of culture media.

### Cell Culture

Spontaneously immortalized retinal Müller cell line MIO-M1 was a generous gift of Dr. Astrid Limb (UCL Institute of Ophthalmology, London, United Kingdom) (Limb et al., 2002). Hepatocellular carcinoma cells Hep3B were purchased from ATCC (Manassas, VA, United States). MIO-M1 and Hep3B cells were maintained in high glucose DMEM (Cleveland Clinic Media Lab, Cleveland, OH, United States) supplemented with 100 units/mL of penicillin, 100 μg/mL of streptomycin and 10% FBS (all from Life Technologies, Carlsbad, CA, United States). Primary human retinal microvascular endothelial cells (REC) were obtained from Cell Systems (Kirkland, WA, United States) and were grown in the fully formulated media from the same provider. All cell cultures were used for experiments at near-confluent density.

### Western Blot Analysis

To detect the extent of HIF stabilization, cells were plated in 60 mm Petri dishes, grown to near-confluency and incubated overnight with the one of the tested drugs at the concentrations indicated in Figure Legends. At the end of the incubation cells were rinsed with ice-cold PBS and harvested by scaring in small volume of RIPA buffer (Sigma-Aldrich, St. Louis, MO, United States) containing protease inhibitor cocktail Complete (Roche, Mannheim, Germany). Cell lysates were briefly sonicated, centrifuged at 20,000 × *g*, and supernatants were used for HIF western blot analysis as essentially described elsewhere (Hoppe et al., 2016).

To detect the *in vivo* potency of HIF stabilizers, the drugs were given by intraperitoneal injection to 8-day old mouse pups (postnatal day 8, P8) who were sacrificed 6 h later by ketamine overdose and their liver, kidney, brain, and retina were dissected and homogenized in RIPA buffer plus Complete using a microtube-fitted pestle. Tissue protein extracts were processed for western blotting the same way as cell cultures described above and reported elsewhere (Hoppe et al., 2014, 2016). All animal procedures here and below were approved by the Cleveland Clinic’s institutional animal care and use committee (IACUC), protocols ## 2016-1677 and 2019-2183.

Hif-1α was detected using rabbit polyclonal antibody from Cayman Chemical (Cat. # 10006421) and HIF-2α was detected using rabbit polyclonal antibody from Novus Biologicals (Cat. # NB100-122).

### ELISA

To determine erythropoietin-(Epo) stimulating potency of the drugs the overnight conditioned media from Hep3B or serum of P8 pups obtained 6 h post-injection by cardiac puncture from an anesthetized animal were subjected to Erythropoietin ELISA (R&D Systems, Minneapolis, MN, United States) according to the manufacturer’s manual. Undiluted conditioned culture media and 1:10 serum dilutions were used.

### Quantitative PCR

Quantitative analysis of mRNA levels was conducted essentially as described earlier (Hoppe et al., 2014, 2016; Singh et al., 2018).Briefly, total RNA extraction was achieved using RNeasy Mini Kit (Qiagen, Germantown, MD, United States). Liver, kidney, brain and retina tissues were collected as described above for protein extracts except the tissue samples were homogenized in RLT buffer containing 1% β-mercaptoethanol and RNA was extracted according to manufacturer instructions. Verso cDNA Synthesis Kit (Thermo Fisher Scientific, Vilnius, Lithuania) was used for reverse transcription to make first stand cDNA. Quantitative PCR was carried out on 7900HT Fast Real-Time PCR System (Applied Biosystems, Foster City, CA, United States) and using QuantiTect SYBR Green PCR kit (Qiagen) together with validated primer sets (QuantiTect Primer Assay, Qiagen). Total volume 20 μL in Applied Biosystems MicroAmp Fast Optical 96-Well Reaction Plate. Cycling conditions: 1. 2 min 50°C; 2. 15 min 95°C; 3. 15 s 94°C; 4. 30 s 55°C; 5. 30 s 72°C; repeat 3–5 39 times. Expression differences were calculated using the delta-delta Ct method.

### Luc-ODD (HIF Reporter) Mouse

Organ-specific HIF stabilization was assayed in the *Gt(ROSA)26Sor^TM 1(Luc)Kael^* (Luc-ODD) mouse strain at age P8 following a 3-h time interval after the injection of a tested drug. Luciferase activity (as a proxy for HIF stability) was detected using Bright-Glo Luciferase Assay System (Promega, Madison, WI, United States) following tissue dissection, homogenization and lysis in Glo Lysis Buffer (Promega) as previously described in detail by our group (Hoppe et al., 2016).

### OIR Model and Retinal Vasculature Analysis

A very well-defined model of ROP (Smith et al., 1994) was utilized for creating oxygen-induced retinopathy in newborn mouse pups by exposing them to 75% oxygen from P7 to P12 and analyzing retinal vasculature at P17 by staining dissected and fixed retina with Alexa568-conjugated Isolectin GS-IB4 from *Griffonia Simplicifolia* (Life Technologies). A detailed procedure for retina dissection, staining, imaging and vascular analysis was published elsewhere (Singh et al., 2019). Testing the drug potency to prevent OIR was carried out according to the standard protocol, i.e., 3 sequential intraperitoneal injections at P6, P8, and P10 as described (Sears et al., 2008). Oxygen-induced retinopathy (vaso-obliteration and neovascularization), was quantified using open-source software available from Xiao et al. (2017). The OIR model uses two standard forms of quantifying retinopathy which are i) capillary drop-out or vasoobliteration or non-perfused retina and (ii) pathological angiogenesis or neovascularization, both established by Smith et al. (1994).

### Müller Cell-Specific *Epas1* Knockout Mouse

To generate conditional knockout, mice carrying *loxP*-flanked *Epas1* (also known as HIF-2α 2-loxP, Stock # 008407, Jackson Laboratory, Bar Harbor, ME, United States) were crossed to hemizygous *Pdgfra-Cre* mice (Stock # 013148, Jackson Lab). Genotyping was carried out by using Jackson Lab-suggested primers and protocol in Hot-Start Tag^TM^ Mastermix (Denville Scientific, Metuchen, NJ, United States). After two rounds of cross-breeding, Epas1^fl/fl^ Cre-negative x Epas1^fl/fl^ hemizygous Cre-positive breeding pairs were used to produced newborn pups for OIR experiment (above). Cre-negative littermates served as controls.

### Statistical Analysis

Statistical analyses of all data (qPCR, bioluminescence of organ lysate from luc-ODD mice, image density for western blot or pixel number quantification for retinal flatmounts) were performed by comparing means using Student’s t-test. We chose to use a paired student’s *t*-test so that we could normalize data to allow for the inherent variation in severity of OIR by splitting litters into 1/2 control PBS injected vs drug injected. In this way, a percentage of pathology (avascular retina and neovascularization) makes an unbiased assessment of drug to control for each compound, and percent reduction in pathology could then be compared across experiments which are performed with multiple litters using different dams and litters sizes (Connor et al., 2009).

The two-tailed probability associated with rejecting the null hypothesis of no difference between observed groups was calculated, using an alpha level of 0.05. Error bars in all figures represent SEM. *P* values were presented as follows, ^∗^ ≤ 0.0001; $ ≤ 0.005; & ≤ 0.01; # ≤ 0.05, and represent statistical difference vs PBS control, unless otherwise indicated as in Figure 5H.

## Data Availability Statement

All datasets presented in this study are included in the article/supplementary material.

## Ethics Statement

The animal study was reviewed and approved by Institutional Animal Care and Use Committee Cleveland Clinic.

## Author Contributions

GH, YB, LM, and JZ performed the experiments. GH and JS conceived the experiments, analyzed the data, and wrote the manuscript. All authors contributed to the article and approved the submitted version.

## Conflict of Interest

The authors declare that the research was conducted in the absence of any commercial or financial relationships that could be construed as a potential conflict of interest.
